# The use of antipsychotic polypharmacy at Ravenswood House Medium Secure Unit: the extent of use and reporting of outcomes

**DOI:** 10.1192/bjo.2021.828

**Published:** 2021-06-18

**Authors:** Laura Cherrington, Hari Patel

**Affiliations:** Ravenswood House Medium Secure Unit

## Abstract

**Aims:**

To evaluate the use of antipsychotic polypharmacy in Ravenswood House Medium Secure Hospital. We also aimed to review the reporting of the outcomes of their use.

**Background:**

The use of antipsychotic polypharmacy (APP) continues to be practised within forensic psychiatric inpatient settings yet there is a lack of robust evidence for the benefits of doing so. The practice is also associated with the use of higher total antipsychotic doses beyond the recommended BNF maximum. Such prescribing is associated with an increased side effect burden. Doctors have a duty to justify the ongoing use of antipsychotic polypharmacy and to avoid potentially ineffective and/or harmful use.

**Method:**

A cross-sectional review of the medication cards for 51 in-patients at Ravenswood House Hospital was completed. Demographic data and data pertaining to diagnoses and medication was also gathered from the electronic patient records.

**Result:**

23 patients (45%) in Ravenswood House Hospital were prescribed antipsychotic polypharmacy. 87% of those prescribed antipsychotic polypharmacy had a primary diagnosis of either schizophrenia or schizoaffective disorder. 19 patients (37%) had two regular antipsychotics prescribed. 74% of these prescriptions were above the recommended BNF maximum. 62% were also prescribed a regular benzodiazepine. The vast majority of indications documented for APP were chronic behavioural disturbance and treatment resistant schizophrenia. The majority of these patients were on a T3. There was a significant under reporting of the rationale of prescribing APP. It could be surmised that at least 11 combinations were in part to mitigate side effects, but only 3 had this documented. There was also a lack of documentation or use of rating scales regarding the clinical outcomes and side effects of APP.

**Conclusion:**

Prescription of antipsychotic polypharmacy is an important issue in secure forensic hospital settings. The lack of clear documentation of clinical effectiveness and side effect burden remains a concern. Wider study is required to establish the benefits of such prescribing to justify its ongoing use.

